# Huge extrahepatic gallstone on percutaneous transhepatic biliary drainage tip—a pediatric case report

**DOI:** 10.1007/s00247-024-06156-4

**Published:** 2025-01-17

**Authors:** Anna-Maria Odenthal, Carsten Meyer, Mark Born, Martin Heimbrodt, Julian Luetkens, Martha Dohna

**Affiliations:** 1 Department of Diagnostic and Interventional Radiology, University Hospital Bonn, Venusberg-Campus 1, 53127 Bonn, Germany; 2 Department of Diagnostic and Interventional Radiology, Pediatric Radiology, University Hospital Bonn, Venusberg-Campus 1, 53127 Bonn, Germany; 3 Department of General Paediatrics, Pediatric Hematology and Oncology, University Hospital Bonn, Bonn, Germany

**Keywords:** Biliary stricture, Cholelithiasis, Lithotripsy, Pediatric liver transplantation, Percutaneous transhepatic biliary drainage

## Abstract

Biliary strictures, which are common in the first year after pediatric liver transplantation, are diagnosed and managed with percutaneous transhepatic cholangiography. In children undergoing percutaneous transhepatic cholangiography, early cholangitis is the most common complication while typical catheter-related complications are obstruction, dislodgement, kinking, or fracture. This case report discusses the unique presentation and management of a 5-year-old girl with an incidental percutaneous transhepatic biliary drainage-associated extrahepatic gallstone formation following treatment of biliary stricture after pediatric liver transplantation. It was effectively treated with percutaneous transhepatic lithotripsy. Although this is a rare but potentially harmful complication, in case of resistance at drainage removal, a drainage-tip stone should be excluded by ultrasound or fluoroscopy.

**Supplementary Information:**

The online version contains supplementary material available at 10.1007/s00247-024-06156-4.

## Introduction

Biliary strictures are a common complication after pediatric liver transplantation. Biliary strictures occur in 4–12% of pediatric liver transplantations due to scarring, usually within 1 year of transplantation, and can be classified as anastomotic strictures (> 80%), extrahepatic or intrahepatic strictures [1]. If left untreated, biliary complications can lead to graft loss due to cholangitis, fibrosis, and graft failure [1]. Clinical signs of biliary strictures include jaundice, pruritus, and acholic stools, while liver biochemistry might show elevated transaminases, gamma-glutamyl transferase, and bilirubin [2]. Elevated serum bile acid may help the diagnostic of early biliary strictures [3]. But clinical assessment of patients can be challenging as signs for biliary strictures may mimic those of graft rejection. Ultrasound is the modality of choice for assessing intra- and extrahepatic biliary ducts. However, due to the high false negative rate of ultrasound examinations in this entity, the diagnostic modalities of choice in biliary strictures include percutaneous transhepatic cholangiography, endoscopic retrograde cholangiopancreatography (ERCP), or magnetic resonance cholangiopancreatography [2]. Treatment of biliary strictures after pediatric liver transplantation includes minimally invasive interventions with percutaneous transhepatic cholangiography combining percutaneous biliary balloon dilatation with percutaneous transhepatic biliary drainage [1]. Other treatment options are ERCP or, less common, surgical revision [1]. The primary aim of percutaneous transhepatic cholangiography is to identify the location and severity of the biliary stricture, and then cross the stricture to place an internal–external drainage. Subsequent procedures include balloon angioplasty and stepwise upsizing of biliary drainage catheters to dilate the stricture [2].

Complications in adults undergoing percutaneous transhepatic cholangiography are rare with 3–10% and can be classified as access-related, non-vascular, vascular, drainage catheter, or stent-related. Most common complications include pain, infection, bile leakage, and catheter occlusion [4]. In children undergoing percutaneous transhepatic cholangiography, drainage dislodgement is the most common complication [1]. Typical catheter-related complications are obstruction, dislodgement, kinking, or fracture [4]. To prevent occlusion, patients and caregivers are trained to frequently flush the drainage catheter [4]. Common guidelines for percutaneous transhepatic cholangiography in pediatric patients are lacking; therefore, it remains unclear how different factors in biliary interventions may cause complications and influence outcomes.

Gallstones are rare in pediatric patients and there are no reported cases of gallstones associated with percutaneous transhepatic biliary drainage in pediatric patients. Gallstones can be classified into cholesterol and pigment stones and are associated with genetic factors, obesity, parenteral nutrition, infection, antibiotic treatment with ceftriaxone, and biliary malformations (choledochal cyst, pancreaticobiliary maljunction) [5]. Furthermore, cholelithiasis is a well-described late toxicity in survivors of childhood cancer, especially after treatment with high doses of platinum, vinca alkaloid, or total body irradiation [5].

A rare cause of biliary stent/drainage obstruction are fungal balls caused by *Candida* species. They might present clinically with obstructive fungal cholangitis and imaging by ERCP or percutaneous transhepatic cholangiography shows filling defects within the stent or intrahepatic bile ducts. Broad-spectrum antifungal medication combined with frequent flushing is supposed to prevent re-obstruction within days as reported by Story et al. [6].

## Case report

Informed consent was obtained from the parents for publishing the case report. We report on a case of a 5-year-old girl who underwent pediatric liver transplantation for hepatoblastoma at the age of 4 years after neoadjuvant treatment with cisplatin, carboplatin, doxorubicin, vincristine, and irinotecan. One month after left-sided split liver transplantation with Roux-en-Y choledochojejunostomy, dilation of the intrahepatic transplant bile ducts was detected by ultrasound in combination with elevated bilirubin, transaminases, and gamma-glutamyl transferase. Ursodeoxycholic acid was therefore commenced. Seven months after pediatric liver transplantation, in September 2023, bilirubin, transaminases, and gamma-glutamyl transferase blood levels significantly increased and scleral icterus was noted. Following ERCP was unsuccessful to pass the Roux-en-Y anastomosis; therefore, a multidisciplinary team decided to perform elective percutaneous transhepatic cholangiography (AlluraClarity angiography system, Philips Healthcare, Amsterdam, Netherlands) to assess the biliodigestive anastomosis and improve biliary drainage. During percutaneous transhepatic cholangiography in October 2023, a benign anastomotic stricture was identified. After balloon angioplasty, an 8.5-French (F) internal–external transhepatic biliary drainage (Biliary Drainage Catheter, Cook Medical, Bloomington, IN) was placed. Postinterventional cholangitis was treated successfully with meropenem. Subsequent balloon angioplasty and stepwise upsizing to a 10-F and then 12-F biliary drainage (PerkuBil® Münchner drainage, Peter Pflugbeil GmbH, Zorneding, Germany) followed without periinterventional complications. With only minor residual stenosis, the 12-F biliary drainage was left in place and follow-up percutaneous transhepatic cholangiography evaluation was recommended 6 months later. The decision to leave the drainage in place for several months was drawn to achieve optimal primary patency and prevent the recurrence of stenosis as recommended by Fang et al. [2]. Regular flushing of the drainage through the parents was recommended. However, most probably due to language barriers, drainage flushing was not or not correctly carried out. Ultrasound follow-up was performed every 4 weeks showing stable minor focal bile duct dilation of the liver transplant. With the placement of the percutaneous transhepatic biliary drainage, the scleral icterus resolved, and bilirubin blood levels progressively normalized.

**Fig. 1:**
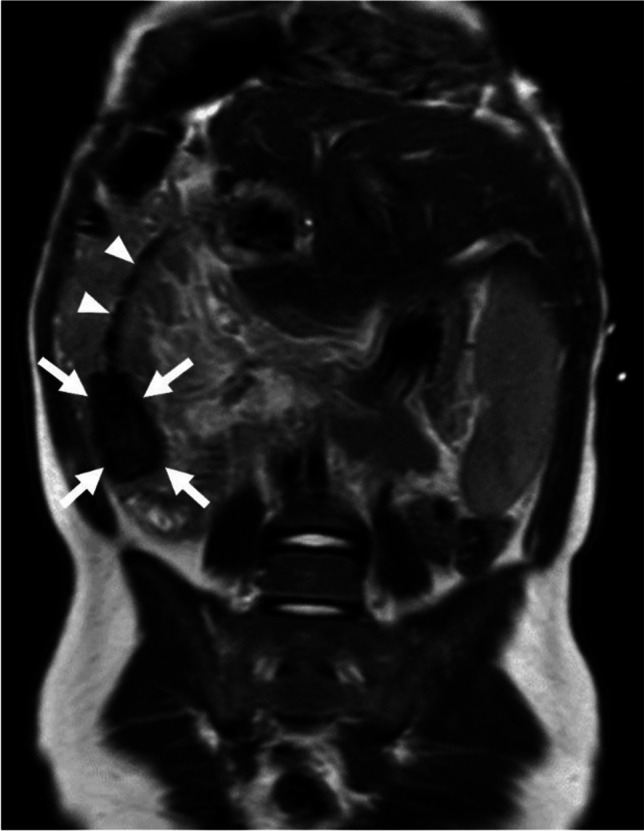
Abdominal magnetic resonance imaging of a 5-year-old girl with a percutaneous transhepatic biliary drainage after liver transplantation. Coronal T2-weighted image shows a hypointense structure (*arrows*) measuring 1.6 × 1.6 × 3.5 cm at the distal end of the biliary drainage (*arrowheads*) in the bowel lumen in the right lower quadrant

In April 2024, during routine follow-up abdominal magnetic resonance imaging (MRI; Ingenia 1.5-T MR-System, Philips Healthcare, Amsterdam, Netherlands) for hepatoblastoma, a cone-shaped T2- and T1-weighted hypointense structure measuring 1.6 × 1.6 × 3.5 cm was detected at the distal end of the percutaneous transhepatic biliary drainage in the bowel lumen indicative of a drainage catheter tip stone (Fig. 1).

**Fig. 2:**
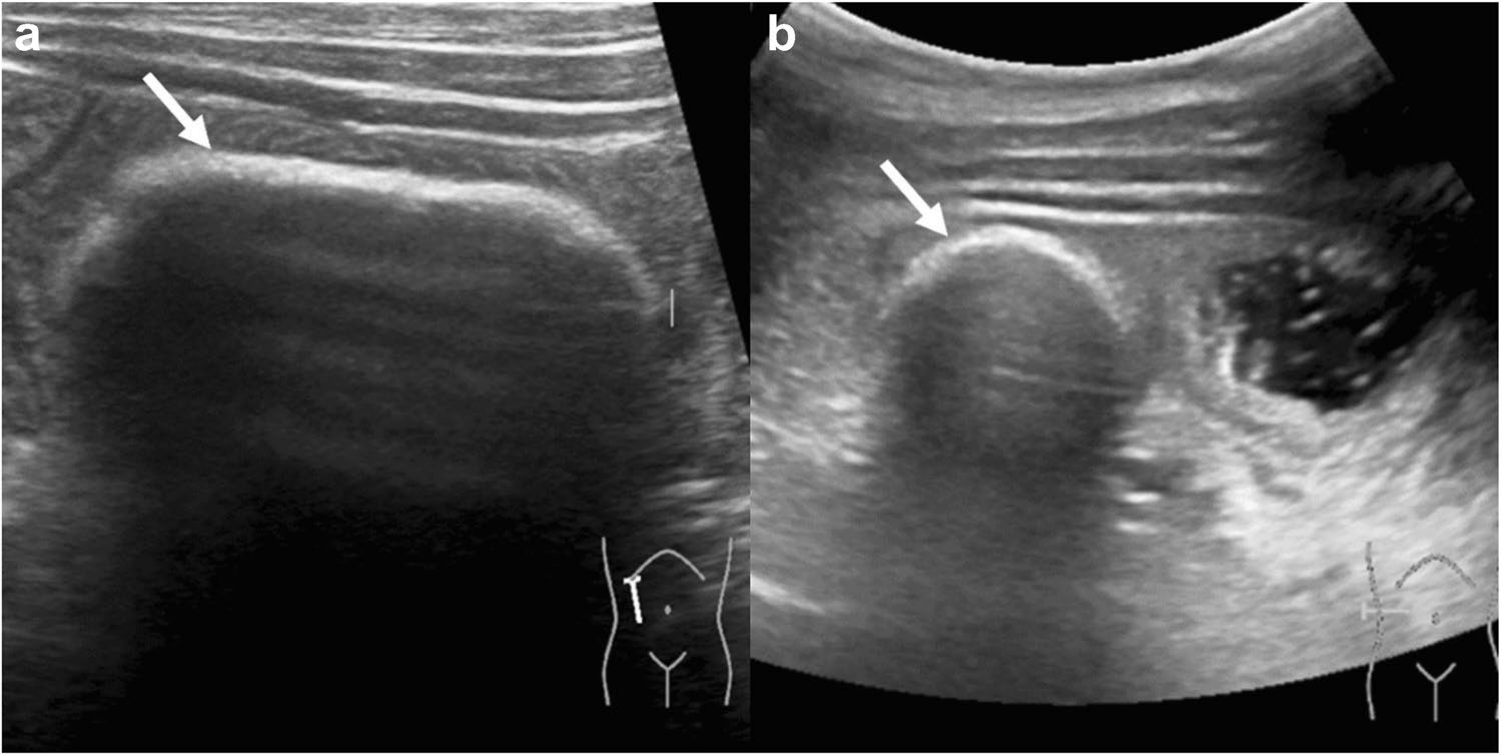
Abdominal ultrasound of a 5-year-old girl with a percutaneous transhepatic biliary drainage with associated stone formation. Abdominal ultrasound was performed to correlate the magnetic resonance imaging finding (see Fig. 1). **a** Longitudinal and (**b**) transverse ultrasound shows the stone at the biliary drainage tip with hyperechoic stone outline (*arrows*) and posterior acoustic shadowing

Correlation with ultrasound (Affiniti 70 Ultrasound, Philips Healthcare, Amsterdam, Netherlands) showed oval acoustic shadowing at the tip of the drainage (Fig. 2) and intermittent short segment small bowel intussusception around the drainage. The stone formation did not cause clinical symptoms, progressive duct dilation, nor hyperbilirubinemia at the time of detection.

**Fig. 3:**
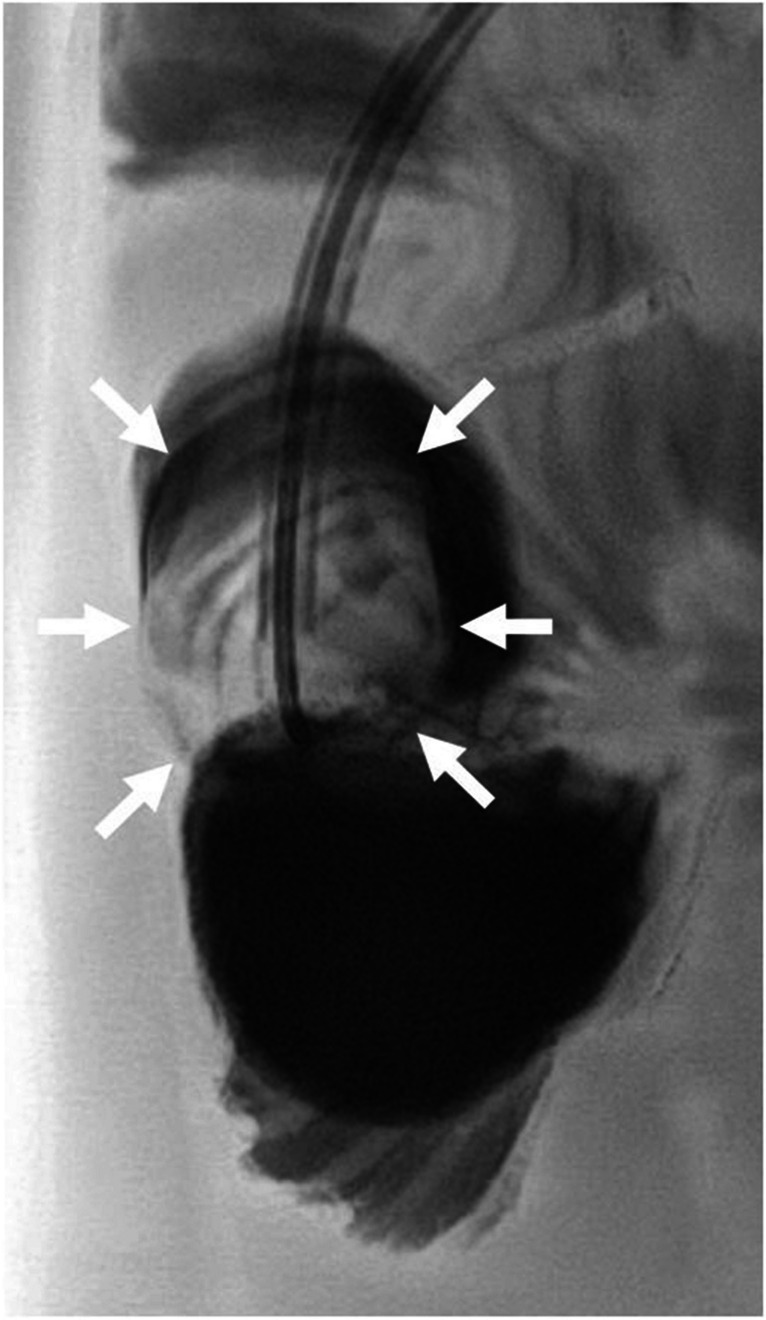
Fluoroscopy of a 5-year-old girl with a percutaneous transhepatic biliary drainage-associated stone formation. Fluoroscopy shows the radiolucent stone (*arrows*) at the drainage tip surrounded by contrast agent. A video is included as Supplementary Material 1

**Fig. 4:**
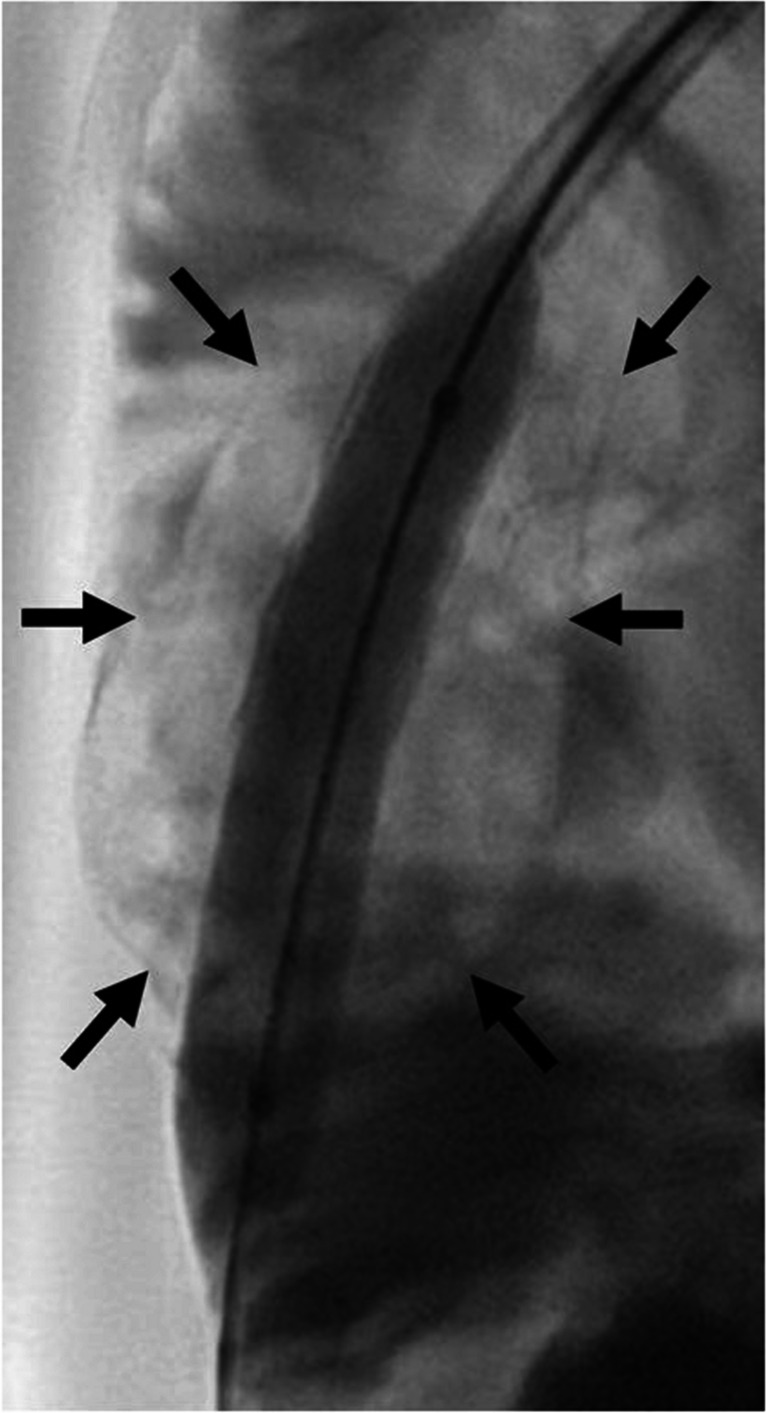
Fluoroscopy of a 5-year-old girl with a percutaneous transhepatic biliary drainage-associated stone formation. Fluoroscopy shows balloon dilatation of the catheter and the radiolucent stone (*arrows*). A video is included as Supplementary Material 2

In a multidisciplinary conference, lithotripsy via the percutaneous transhepatic biliary drainage was chosen over surgery as the least invasive treatment strategy with the option to salvage the percutaneous transhepatic cholangiography pathway in case of residual stenosis. In May 2024, after fluoroscopic identification of the radiolucent stone surrounded by contrast agent in the bowel lumen (Fig. 3 and Supplementary Material 1), percutaneous transhepatic lithotripsy was performed via the percutaneous transhepatic biliary drainage with an 8-mm and then a 12-mm balloon (Mustang™ Balloon Dilatation Catheter, Boston Scientific, Marlborough, MA). The stone appeared to be somewhat elastic and flexible expanding during balloon dilatation (Fig. 4 and Supplementary Material 2).

**Fig. 5:**
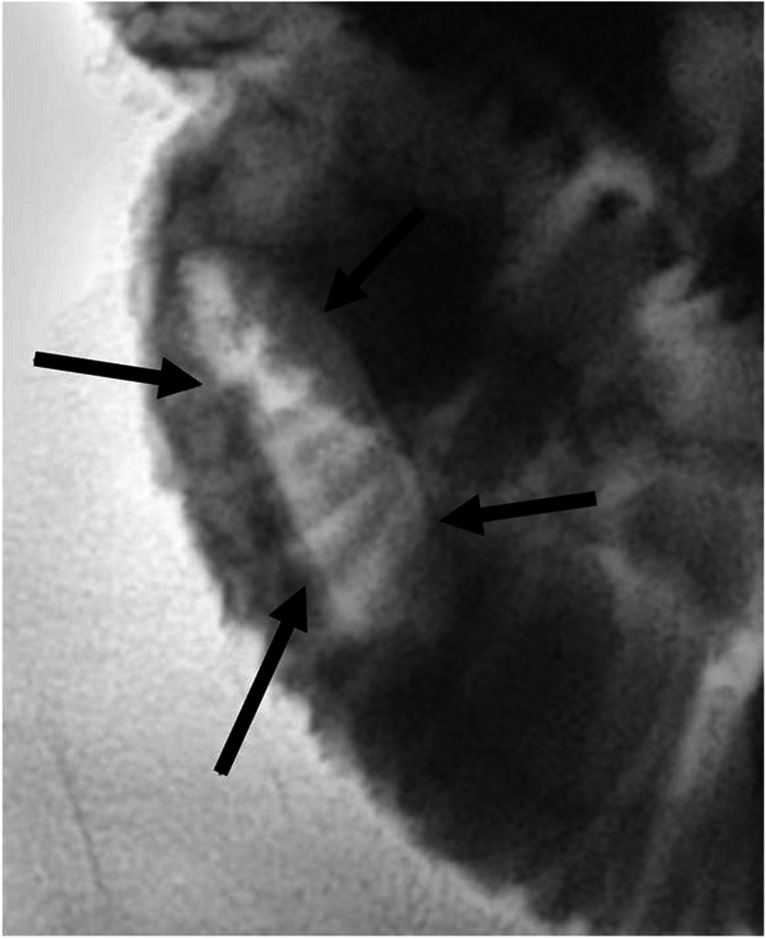
Fluoroscopy of a 5-year-old girl with a percutaneous transhepatic biliary drainage-associated stone formation. Displaced radiolucent stone fragments (*arrows*) are visible in the small bowel lumen in the lower right quadrant after successful lithotripsy. A video is included as Supplementary Material 3

During the procedure, the stone then suddenly dislodged from the drainage tip and several larger fragments were visible in the small bowel confirming rupture of the stone (Fig. 5 and Supplementary Material 3).

Additional ultrasound control showed the drainage tip free from stone, confirming successful lithotripsy. The biliary drainage was replaced with a 5-F catheter (Beacon® Tip 5.0 Fr Angiographic Catheter, Cook Medical, Bloomington, IN) as a placeholder without drainage function in case of postinterventional complications. There were no intervention-associated complications such as gallstone ileus, infection, or bleeding. During the last percutaneous transhepatic cholangiography evaluation 7 weeks later, the 5-F catheter was removed as successful stricture resolution with a normal biliary drainage time and regular liver biochemistry was confirmed. Unfortunately, due to language barriers, the gallstone fragments have not been retrieved and thus could not be further analyzed.

## Discussion

Biliary complications after pediatric liver transplantation are a common cause of morbidity. The case of a 5-year-old girl who underwent pediatric liver transplantation and neoadjuvant chemotherapy for hepatoblastoma highlights the complexity and challenges of managing biliary complications after pediatric liver transplantation. While occlusion of a percutaneous transhepatic biliary drainage is a common complication which can be prevented by regular saline flushing, in our patient, an unexpected complication occurred with large gallstone formation at the drainage tip possibly due to lack of regular flushing of the drainage. This stone was incidentally detected on follow-up imaging 4 months after drainage placement and not associated with obstruction, clinical symptoms, or elevated liver biochemistry. Fortunately, the stone was detected and injury of the liver transplant or gallstone ileus after drainage removal could be prevented. Although in this case the diagnosis was made by MRI, ultrasound, which is easy and fast to perform, is perfectly adequate to detect or possibly rule out a catheter/stone complex in pediatric patients. Of note, the stone in our patient was radiolucent and could easily have been overseen during fluoroscopy or abdominal radiograph.

The prevalence of percutaneous transhepatic biliary drainage-associated lithiasis is unclear, as the stone may dislocate from the tip at removal but nevertheless cause organ injury. This might be misinterpreted as intervention-related tissue/organ injury or bleeding. If left undiagnosed, such stones can potentially cause ileus, intussusception, bile duct rupture, or haemobilia. Othman et al. reported on an adult patient with gallstone ileus and biliodigestive anastomosis perforation after retrieval of a percutaneous transhepatic biliary drainage with an attached biliary stone [7]. Chaouch et al. reported on a case of a forgotten plastic biliary stent in an adult patient that led to a stent/stone complex, a “stentolith,” 9 years later [8]. However, it remains unclear if an earlier exchange of the drainage and/or regular flushing of the drainage might have prevented stone formation. As the fragments have not been retrieved, a fungal infection as a differential cause could not be ruled out, although our patient did not present with signs of infection nor obstruction. In addition, other factors like inflammation, chemotherapy with platinum derivates and vincristine, antibiotics, or immunosuppression due to liver transplant might have contributed to stone formation in this patient.

This case demonstrates the importance of careful follow-up and invites clinicians to actively think of extrahepatic biliary stones at the tip of biliary drainages. In case of resistance at catheter removal, forced retraction against resistance should be avoided and an ultrasound or fluoroscopy with contrast agent should be carried out to rule out radiolucent stone formation at the drainage catheter tip. This case illustrates the importance of pediatric radiologic expertise not only concerning the diagnostics of biliary strictures but also in treating patients with percutaneous transhepatic biliary drainage with a tailored interventional approach.

## Supplementary Information

Below is the link to the electronic supplementary material.

Supplementary file1 (MP4 17931 KB)

Supplementary file2 (MP4 10897 KB)

Supplementary file3 (MP4 11638 KB)

## Data Availability

No datasets were generated or analysed during the current study.
